# Assessment of the effect of age on macular layer thickness in a healthy Chinese cohort using spectral-domain optical coherence tomography

**DOI:** 10.1186/s12886-018-0842-y

**Published:** 2018-07-11

**Authors:** Qian Xu, Ying Li, Ying Cheng, Yi Qu

**Affiliations:** 1 Department of Geriatrics, Qilu Hospital of Shandong University, No. 107, Wenhuaxi Road, Jinan, 250012 Shandong China; 2 Department of Ophthalmology, The Central Hospital of Taian, Tai’an, 271000 Shandong China

**Keywords:** SD-OCT, Aging, Retina, Choroid, Layer thickness, Macula

## Abstract

**Background:**

To determine the effect of age on the thickness of individual retinal and choroidal vascular layers in the macula in an ophthalmologically healthy Chinese cohort by using spectral-domain optical coherence tomography (SD-OCT).

**Methods:**

In all, 525 health eyes of 525 subjects were examined with SD-OCT. The instrument automatically obtained the regional retinal thickness of 8 layers. Subfoveal choroidal vascular layers’ thickness was measured using enhanced depth imaging mode. The correlation of age with layer thickness measurements was determined.

**Results:**

No age-associated variation was found on retinal thickness (RT) in the fovea; however, the foveal thickness of outer nuclear layer (ONL), retinal pigment epithelium (RPE) and vascular sublayers of the choroid decreased significantly with aging in this area (*P* < 0.05, respectively). Significant age-related reduction was seen in RT in the pericentral and peripheral rings (*P* < 0.05, respectively). The significant variation in thinning of the ganglion cell layer, inner plexiform layer, and ONL with aging is thought to be the main determinant of these results (*P* < 0.05, respectively). On the contrary, the RPE layout showed age-related thickening (P < 0.05, respectively) in the pericentral and peripheral regions.

**Conclusions:**

The thickness of individual layers of the macula may be determinants of the age-related variations observed in the ophthalmologically healthy Chinese cohort, as assessed by SD-OCT examination.

## Background

Detailed assessment of the macular area is critical in the diagnosis and management of a variety of ocular diseases. Traditional investigations such as fundus photography and fluorescein angiography can only provide qualitative and prospective information, therefore being subjective and relatively insensitive to small changes of the macula and unable to provide any cross-sectional or thickness-related data. The introduction of optical coherence tomography (OCT) has made it possible to noninvasively quantify macular structures in vivo with high resolution [1, 2]. In addition, because OCT is easy to use, ensures patient comfort, and is economical, it has become an important diagnostic tool for fundus diseases.

Spectral domain-OCT (SD-OCT) is an advanced modification of time-domain OCT that provides better reproducibility for image acquisition, high-resolution three-dimensional images, and volumetric analyses [3, 4]. Techniques such as enhanced-depth imaging (EDI) permit improved analysis of the living choroid [5]. In addition, advances in layer segmentation algorithms have facilitated the automatic measurement of the thickness of individual retinal layers [6, 7].

Thickness measurement of the macula using SD-OCT has been shown to play an important role in understanding of the anatomy of individual macular layers, each of which has its own normal three-dimensional shape and may be affected in various ways by different diseases. Several studies have investigated morphological abnormalities of the macula in some ocular diseases by using SD-OCT. Macular thickening due to fluid accumulation is found in diabetic retinopathy and central serous chorioretinopathy (CSCR) [8–10]. The visual acuity of center-involved diabetic macular edema or CSCR eyes may be dependent on the disorganization of the retinal inner layers or the outer nuclear layer (ONL) in the fovea [8, 9]. Macular morphology is also an important parameter for monitoring and staging of glaucoma or age-related macular degeneration (AMD) [11, 12]. Moreover, clinically detected morphologic changes of different retinal layers were identified in many systemic diseases such as multiple sclerosis, [13] Parkinson’s disease, [14] Alzheimer’s disease, [15] and diabetes mellitus with preclinical retinopathy [16]. Therefore, measuring macular thickness by OCT is a powerful tool for physicians to evaluate progression of certain diseases, especially those that involve certain layers.

Recently, SD-OCT was used to study normal retinal and choroid thickness among subjects of different ethnicities, gender, and ages [17–20]. Age-related reduction in macular thickness was shown in a Caucasian and a Japanese population [18, 21]. However, the aforementioned reports were insufficient to facilitate the detailed analysis of the structure of specific retinal and choroidal layers. Moreover, to our best knowledge, there is no normative database available for the thickness of individual macular layers in the Chinese population.

Therefore, in this study, we used SD-OCT to measure the total retinal thickness (RT), the thickness of individual retinal layers of the macula that were divided into nine sectors, and the subfoveal choroidal thickness (SFCT) including vascular sublayers in 525 ophthalmologically healthy eyes in order to evaluate the effect of age on normal mean regional retinal and subfoveal choroidal layers on the macula.

## Methods

### Subjects

In this prospective observational study, self-reported, ophthalmologically healthy subjects of Chinese ethnicity aged ≥20 years were randomly recruited from May 2015 to December 2016. The study adhered to the tenets of the Declaration of Helsinki and was approved by the Ethics Committee of Qilu Hospital. Written consent was obtained from each subject.

All participants underwent a comprehensive ophthalmologic examination including best corrected visual acuity (BCVA), refraction, slit-lamp biomicroscopy, intraocular pressure (IOP) measurement by Goldmann applanation tonometry, and fundus photography obtained by two trained ophthalmologists. The inclusion criteria were as follows: BCVA≥20/25 Snellen (0.1 LogMAR), spherical equivalent refractive error not exceeding ±6.0 diopters, IOP < 21 mm Hg, no history of any ocular abnormalities other than mild to moderate cataracts, no family history of glaucoma, and no systemic diseases such as hypertension, diabetes, or any other autoimmune or infectious diseases. One eye of each participant was randomly selected for OCT examination with the pupil dilated using 0.1% tropicamide.

### Optical coherence tomography and layer segmentation

OCT measurements were performed with the Heidelberg Spectralis OCT (Heidelberg Engineering, Heidelberg, Germany). The instrument incorporates a real-time eye-tracking system that combines a confocal scanning laser ophthalmoscope and SD-OCT scanners to adjust for eye motion. The experienced operators performed all OCT scans under the same intensity of dim room lighting. If any scan was of insufficient quality, it was immediately repeated and reviewed until the image was satisfactory.

**Fig. 1:**
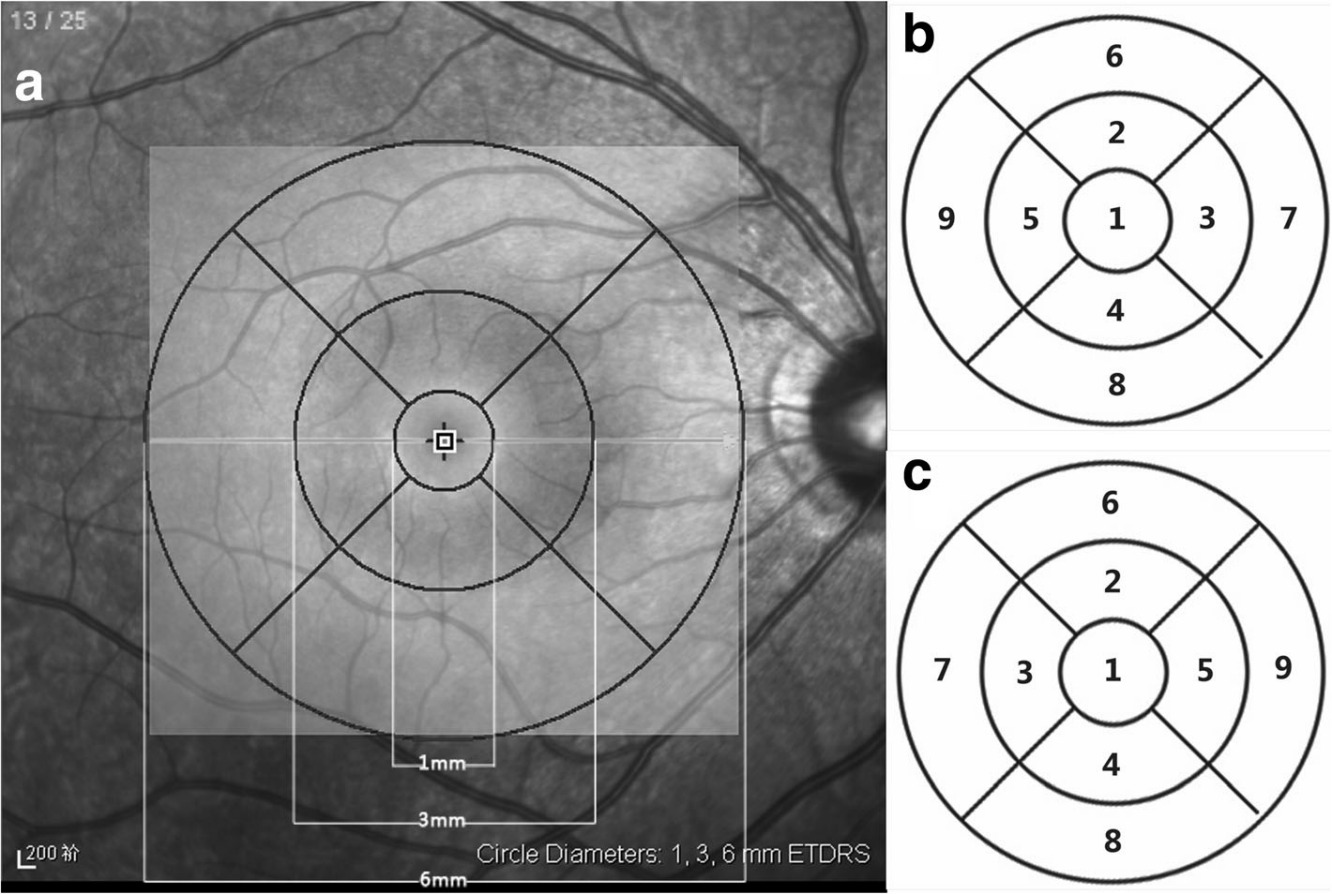
Early Treatment Diabetic Retinopathy Study (ETDRS) grid. **a** Delineation of the nine macular sectors, according to the ETDRS, within which we measured macular layer thickness. **b** Nine ETDRS sectors in Right eye. **c** Nine ETDRS sectors in Left eye

**Fig. 2:**
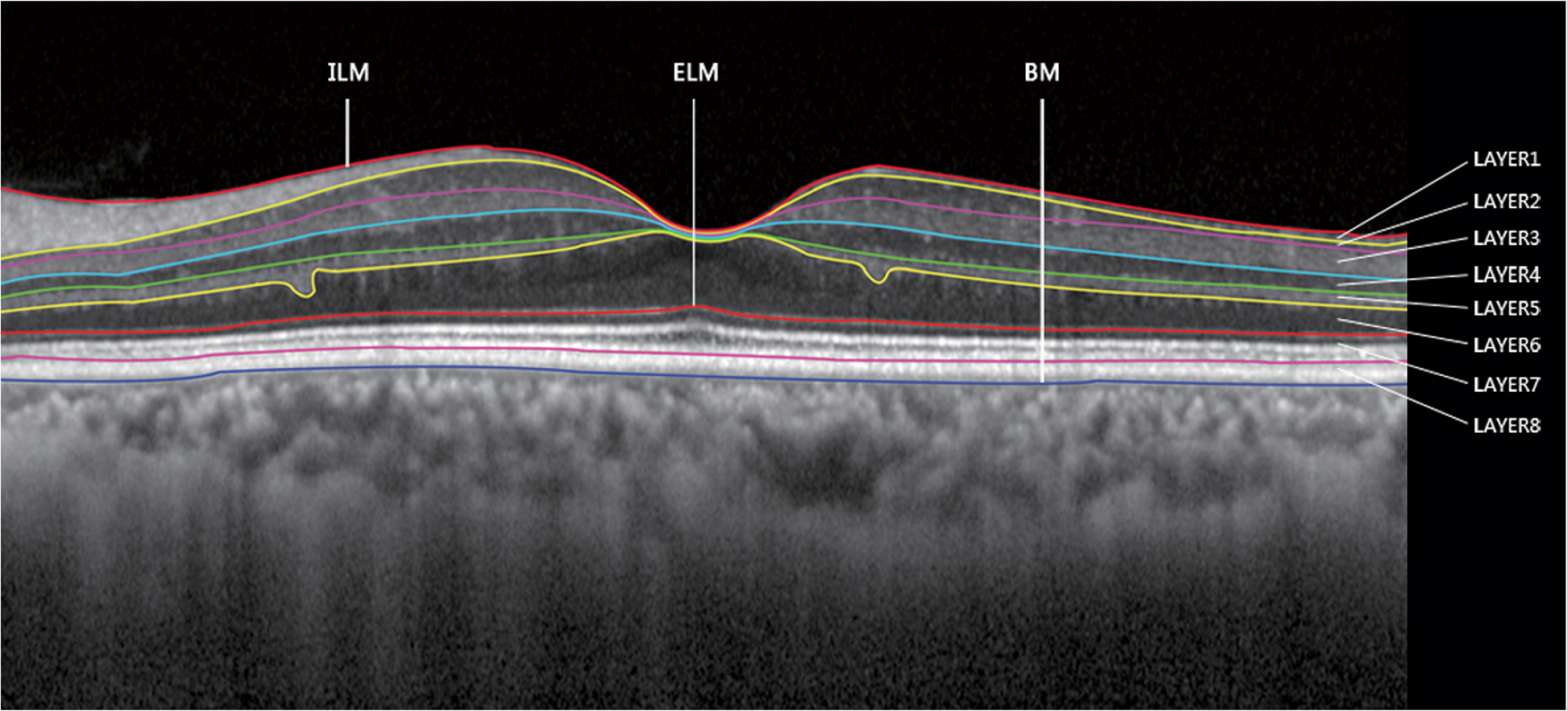
An automated method (with manual correction) was used to segment retinal boundaries in each of the averaged B-scans in the spectral-domain optical coherence tomography examination. The individual retinal layers were identified as follows (from inner to outer surface): (Layer 1) Retinal nerve fiber layer (RNFL), (Layer 2) Ganglion cell layer (GCL), (Layer 3) Inner plexiform layer (IPL), (Layer 4) Inner nuclear layer (INL), (Layer 5) Outer plexiform layer (OPL), (Layer 6) Outer nuclear layer (ONL), (Layer 7) Photoreceptor layers (PRL), (Layer 8) Retinal pigment epithelium (RPE). Abbreviations: ILM: internal limiting membrane, BM: Bruch’s membrane, ELM: external limiting membrane

The macula was segmented into three concentric circles with diameters of 1 mm, 3 mm, and 6 mm, which were termed as the fovea, pericentral ring, and peripheral ring, respectively (Fig. 1a). Furthermore, the pericentral and peripheral rings were equally divided into four regions: superior, nasal, inferior, and temporal, according to the Early Treatment Diabetic Retinopathy Study (ETDRS). In all, 9 sectors were involved in the macular area (Fig. 1b and c). Each SD-OCT image was analyzed using an image segmentation algorithm, and thickness profiles of RT and eight individual retinal layers were automatically generated by the Spectralis OCT software (Fig. 2). The distance from the internal limiting membrane to the outer border of Bruch’s membrane or external limiting membrane was taken as the RT or inner retinal thickness (IRT), and the individual retinal layers were identified as follows (from inner to outer surface): retinal nerve fiber layer (RNFL), ganglion cell layer (GCL), inner plexiform layer (IPL), inner nuclear layer (INL), outer plexiform layer (OPL), ONL, photoreceptor layer with retinal pigment epithelium (PRL + RPE), and the RPE alone.

**Fig. 3:**
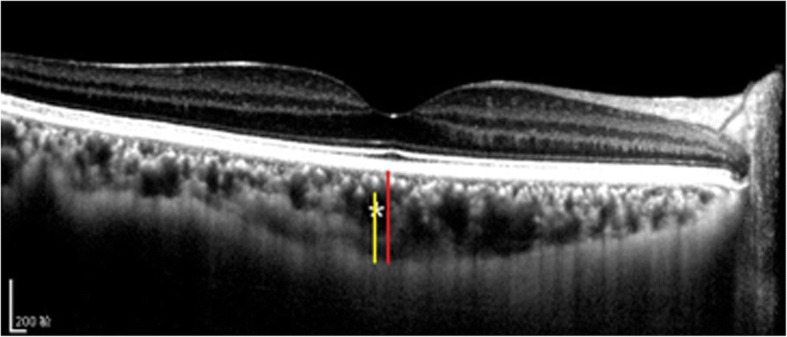
Choroidal vasculature measurements. The vertical red bars delineate the subfoveal choroidal thickness from the retinal pigment epithelium to the choroido-scleral interface in the fovea. The yellow bars delineate the Haller’s layer was measured from the inner border of the choroido-scleral interface to the innermost point of the selected large choroidal vessel. Asterisk is example of large choroidal vessel

SFCT was determined from images acquired by the Heidelberg Spectralis OCT device with enabled EDI mode and analyzed with the OCT-supplied software (Fig. 3). High-quality horizontal and vertical line scans centered on the fovea were obtained. In the fovea, the SFCT was manually measured from the hyperreflective line of the Bruch’s membrane to the innermost surface of the choroido-scleral interface [5]. The thickness of Haller’s layer was measured from the inner border of the choroido-scleral interface junction to the innermost point of the selected large choroidal vessel that was located close to the choroido-scleral border and within the closest proximity to the locations of the choroidal thickness measurement lines. The difference of these measurements was considered as the depth of the choriocapillaris/Sattler’s layer [10]. Means were calculated as the average thicknesses measured from horizontal and vertical sections.

### Statistical analysis

All data were described as mean ± standard deviation (SD) where applicable. Statistical analyses were performed with commercial statistical software (IBM SPSS Statistics 21; SPSS Inc., Chicago, IL). The partial correlation test was used to determine the effect of age on individual layers’ thicknesses with spherical equivalent and IOP as confounders that were known to influence OCT thickness measurements [17, 22]. Finally, simple linear regression analysis was performed for the layer whose thickness correlated significantly with age. *P* values < 0.05 were considered statistically significant.

## Results

The study included 525 ophthalmologic healthy eyes of 525 subjects ranging in age from 20 to 87 years (mean age, 44.82 ± 17.74 years). Demographic and ocular features of the study population are presented in Table 1.

**Table 1:** Demographic and Ocular Features of Included Subjects

Age groups (y)	Number	Men/Women (ratio)	Mean Refractive Error (diopters)	Mean Intraocular Pressure (mm Hg)	Mean Age (y)
20–29	176	90/86(1.05)	−1.93 ± 1.71	14.1 ± 2.4	25.71 ± 1.52
30–39	50	27/23(1.17)	−0.74 ± 1.19	14.3 ± 2.1	35.38 ± 3.13
40–49	108	68/40(1.7)	−0.36 ± 1.29	13.8 ± 2.3	46.07 ± 2.33
50–59	79	41/38(1.08)	−0.54 ± 1.54	14.6 ± 2.1	53.90 ± 2.51
60–69	60	32/28(1.14)	0.08 ± 0.59	14.5 ± 2.6	64.53 ± 3.12
70+	52	27/25(1.08)	−0.04 ± 0.91	14.2 ± 2.0	79.40 ± 5.25
Total	525	285/240(1.19)	−0.87 ± 1.60	14.3 ± 2.3	44.82 ± 17.74

Mean refers to mean ± standard deviation

The mean thickness of RT and eight individual retinal layers in 9 macular EDTRS sectors of all participants are presented in Appendix: Table 6. IRT was excluded owing to similar results as that of RT (data not shown). After adjusting for spherical equivalence and IOP, no significant correlation was found on foveal RT (*P* = 0.54) (Table 2). In the fovea, the ONL and RPE correlated negatively with age (Correlation = − 0.15, *P* < 0.01; Correlation = − 0.09, *P* = 0.03, respectively) (Table 2); however, the RNFL, INL, and OPL correlated positively with age (Correlation = 0.13, 0.30, 0.10, respectively; all *P* < 0.05) (Table 2). Regression analysis indicated an increase for the RNFL boundary as well as the INL boundary and a loss for the RPE boundary with increasing age (Beta = 0.13, 0.10, − 0.14, respectively; all *P* < 0.05) (Table 2).

**Table 2:** Correlations of Age with Regional Retinal Thickness of Foveal Layers

Retinal Layer	RT	RNFL	GCL	IPL	INL	OPL	ONL	PRL + RPE	RPE
*P* Value^a^	0.54	< 0.01^c^	0.36	0.10	< 0.01^c^	0.02^c^	< 0.01^c^	0.77	0.03^c^
Correlation^a^	0.03	0.13	− 0.04	0.07	0.30	0.10	−0.15	− 0.01	− 0.09
*P* Value^b^	0.28	< 0.01^c^	0.15	0.25	< 0.01^c^	0.63	0.07	0.68	< 0.01^c^
Beta^b^	0.05	0.13	− 0.06	0.05	0.10	0.02	−0.08	−0.02	− 0.14

*Abbreviations*: *RT* Retinal thickness, *RNFL* Retinal nerve fiber layer, *GCL* Ganglion cell layer, *IPL* Inner plexiform layer, *INL* Inner nuclear layer, *OPL* Outer plexiform layer, *ONL* Outer nuclear layer, *PRL* Photoreceptor layer, *RPE* Retinal pigment epithelium, *IOP* Intraocular pressure

^a^Partial correlation coefficient after adjusting for spherical equivalent and IOP

^b^Simple linear regression analysis

^c^Statistically significant

As shown in Table 3, the total SFCT, thickness of the large choroidal vessel layer (Haller’ s layer), choriocapillaris layer and Sattler’ s layer (medium choroidal vessel layer) in the fovea showed significant negative correlation with age (Correlation = − 0.55, − 0.42, − 0.46, respectively; all *P* < 0.05). In addition, our study found that SFCT and the thickness of choroidal vascular sublayers decreased linearly with age (Beta = − 0.61, − 0.47, − 0.52, respectively) (all *P* < 0.05).

**Table 3:** Correlations of Age with Thickness of Suboveal Choroidal Layers

Suboveal Choroidal Layer	Thickness (mean ± SD, μm)	*P* Value^a^	Correlation^a^	*P* Value^c^	Beta^c^
Total Choroidal Thickness	225.02 ± 35.71	< 0.01^b^	− 0.55	< 0.01^b^	− 0.61
Haller’s Layer Thickness	157.62 ± 26.57	< 0.01^b^	− 0.42	< 0.01^b^	− 0.47
Choriocapillaris /Sattler’s layer Thickness	67.41 ± 17.83	< 0.01^b^	− 0.46	< 0.01^b^	− 0.52

^a^Partial correlation coefficient after adjusting for spherical equivalent and IOP

^b^Statistically significant

^c^Simple linear regression analysis

Significant age-related reductions were seen for the RT, GCL, and IPL in the pericentral and peripheral rings (all *P* < 0.05; Tables 4 and 5). Moreover, the OPL of the temporal sector, ONL except the temporal sector in the pericentral ring (both *P* < 0.05; Table 4), RNFL of both superior and inferior sectors, INL except the superior sector, ONL of all sectors, and PRL + RPE of the inferior sector in the peripheral ring (all *P* < 0.05; Table 5) showed significant decreases with respect to age. However, significant age-related increase was demonstrated in the RNFL of the temporal sector, INL and OPL of the nasal sector, RPE of all sectors in the pericentral ring (all *P* < 0.05; Table 4), RNFL of the temporal sector, OPL of the nasal sector, and RPE of the superior and temporal sectors in the peripheral ring (all *P* < 0.05; Table 5).

**Table 4:** Correlations of Age with Thickness of Macular Retinal Layers in Sectors of the Pericentral ring

Retinal Layer	Pericentral Superior				Pericentral Nasal				Pericentral Inferior				Pericentral Temporal			
	*P* Value^a^	Correlation^a^	*P* Value^b^	Beta^b^	*P* Value^a^	Correlation^a^	*P* Value^b^	Beta^b^	*P* Value^a^	Correlation^a^	*P* Value^b^	Beta^b^	*P* Value^a^	Correlation^a^	*P* Value^b^	Beta^b^
RT	< 0.01^c^	− 0.27	< 0.01^c^	− 0.24	<.01^c^	−.21	< 0.01^c^	− 0.16	< 0.01^c^	−.26	<.01^c^	−.23	<.01^c^	− 0.25	< 0.01^c^	− 0.19
RNFL	0.72	− 0.02	0.51	− 0.03	0.74	0.01	0.66	0.02	0.17	−0.06	0.27	−0.05	< 0.01^c^	0.24	< 0.01^c^	0.31
GCL	< 0.01^c^	− 0.31	< 0.01^c^	− 0.32	< 0.01^c^	− 0.24	< 0.01^c^	− 0.24	< 0.01^c^	− 0.32	< 0.01^c^	− 0.34	< 0.01^c^	− 0.35	< 0.01^c^	− 0.37
IPL	< 0.01^c^	− 0.37	< 0.01^c^	− 0.39	< 0.01^c^	− 0.33	< 0.01^c^	− 0.33	< 0.01^c^	− 0.36	< 0.01^c^	− 0.37	< 0.01^c^	− 0.28	< 0.01^c^	− 0.29
INL	0.60	− 0.02	0.20	0.06	< 0.01^c^	0.18	< 0.01^c^	0.27	0.29	− 0.05	0.75	− 0.01	0.08	−0.08	1.00	0.00
OPL	0.63	0.02	0.08	0.08	< 0.01^c^	0.18	< 0.01^c^	0.19	0.49	0.03	0.85	−0.01	< 0.01^c^	− 0.13	< 0.01^c^	− 0.19
ONL	< 0.01^c^	− 0.13	< 0.01^c^	− 0.14	< 0.01^c^	− 0.28	< 0.01^c^	− 0.26	0.01^c^	− 0.11	0.36	−0.04	0.27	−0.05	0.39	0.04
PRL + RPE	0.16	0.06	0.31	0.01	0.91	−0.01	0.64	0.02	0.76	0.01	0.40	0.04	0.18	0.06	0.32	0.09
RPE	< 0.01^c^	0.19	< 0.01^c^	0.23	0.01^c^	0.12	0.01^c^	0.11	<.01^c^	.18	<.01^c^	0.23	< 0.01^c^	0.20	< 0.01^c^	0.21

*Abbreviations*: *RT* Retinal thickness, *RNFL* Retinal nerve fiber layer, *GCL* Ganglion cell layer, *IPL* Inner plexiform layer, *INL* Inner nuclear layer, *OPL* Outer plexiform layer, *ONL* Outer nuclear layer, *PRL* Photoreceptor layer, *RPE* Retinal pigment epithelium; IOP, Intraocular pressure

^a^Partial correlation coefficient after adjusting for spherical equivalent and IOP

^b^Simple linear regression analysis

^c^Statistically significant

**Table 5:** Correlations of Age with Thickness of Macular Retinal Layers in Sectors of the Peripheral ring

Retinal Layer	Pericentral Superior				Pericentral Nasal				Pericentral Inferior				Pericentral Temporal			
	*P* Value^a^	Correlation^a^	*P* Value^b^	Beta^b^	*P* Value^a^	Correlation^a^	*P* Value^b^	Beta^b^	*P* Value^a^	Correlation^a^	*P* Value^b^	Beta^b^	*P* Value^a^	Correlation^a^	*P* Value^b^	Beta^b^
RT	< 0.01^c^	− 0.37	< 0.01^c^	− 0.37	< 0.01^c^	− 0.23	< 0.01^c^	− 0.22	< 0.01^c^	− 0.31	< 0.01^c^	− 0.32	< 0.01^c^	− 0.28	< 0.01^c^	− 0.25
RNFL	0.01^c^	− 0.11	< 0.01^c^	− 0.18	0.19	−0.06	< 0.01^c^	− 0.12	0.02^c^	− 0.10	< 0.01^c^	− 0.15	0.01^c^	0.11	< 0.01^c^	0.15
GCL	< 0.01^c^	− 0.39	< 0.01^c^	− 0.37	< 0.01^c^	− 0.31	< 0.01^c^	− 0.29	< 0.01^c^	− 0.32	< 0.01^c^	− 0.33	< 0.01^c^	− 0.36	< 0.01^c^	− 0.36
IPL	< 0.01^c^	− 0.40	< 0.01^c^	− 0.40	< 0.01^c^	− 0.35	< 0.01^c^	− 0.35	< 0.01^c^	− 0.26	< 0.01^c^	− 0.28	< 0.01^c^	− 0.28	< 0.01^c^	− 0.27
INL	0.08	− 0.16	0.01^c^	− 0.11	< 0.01^c^	− 0.18	< 0.01^c^	− 0.18	< 0.01^c^	− 0.17	< 0.01^c^	− 0.14	< 0.01^c^	− 0.33	< 0.01^c^	− 0.29
OPL	0.33	0.04	0.02^c^	0.11	< 0.01^c^	0.23	< 0.01^c^	0.30	0.36	0.04	0.29	0.05	0.41	−0.04	0.77	0.01
ONL	< 0.01^c^	− 0.28	< 0.01^c^	− 0.29	< 0.01^c^	− 0.36	< 0.01^c^	− 0.39	< 0.01^c^	− 0.18	< 0.01^c^	− 0.16	< 0.01^c^	− 0.25	< 0.01^c^	− 0.24
PRL + RPE	0.87	− 0.01	0.59	0.02	0.10	−0.07	0.25	−0.05	0.04^c^	− 0.09	0.05	−0.09	0.34	0.04	0.03	0.10
RPE	0.04^c^	0.09	< 0.01^c^	0.16	0.45	0.03	0.10	0.07	0.80	0.01	0.22	0.05	< 0.01c	0.20	< 0.01^c^	0.24

*Abbreviations*: *RT* Retinal thickness, *RNFL* Retinal nerve fiber layer, *GCL* Ganglion cell layer, *IPL* Inner plexiform layer, *INL* Inner nuclear layer, *OPL* Outer plexiform layer, *ONL* Outer nuclear layer, *PRL* Photoreceptor layer, *RPE* Retinal pigment epithelium, *IOP* Intraocular pressure

^a^Partial correlation coefficient after adjusting for spherical equivalent and IOP

^b^Simple linear regression analysis

^c^Statistically significant

## Discussion

In this study, consistent with previous reports, [23–25] no significant correlation was found between age and foveal RT. However, the ONL, RPE and choroid vascular sublayers in this region showed significant age-related thinning, accompanied with age-related thickness of RNFL, INL, and OPL. To our best knowledge, we believe this is the first study to report the detailed age-related changes of foveal microstructure. On comparing with total thickness, assessment of macular layers provides a higher diagnostic power. The most significant observation herein was the age-related thinning of foveal ONL, RPE and choroid measurements even in ophthalmologically healthy subjects, which could be a potential anatomic predisposing factor for monitoring the age-related diseases in this eye region. The atrophy of RPE and choroid layer in the central retina is a feature of early/intermediate AMD, the incidence of which is increased with age. A 32% loss in the RPE/PRL thickness and a 22% loss in ONL thickness were found over the drusen as compared to the adjacent drusen-free regions in AMD patients [26]. The choriocapillaris degenerates in early stages of AMD, before loss of photoreceptor cells or RPE [12]. Although AMD is a complicated process that involves both age-related change and tissue damage caused by multiple stresses, age plays the most important role [27]. Functionally normal RPE and choroidal vasculature play a critical role in maintaining retinal health. Thinner RPE and choroid layer thickness may be anatomic features lead to increased risk in AMD. Our results showed that assessment of the foveal layer thickness with OCT in ophthalmologically healthy aged subjects’ eyes may lead to early identification and treatment of AMD. Moreover, further investigations are needed on the mechanism of age-related variations of the ONL, RPE, RNFL, INL, and OPL in the fovea.

We have observed significant age-associated reductions of RT in the pericentral and peripheral rings that were distinct from the foveal results; these results were consistent with previous studies [19, 28, 29]. Notably, age-related changes of GCL, IPL, and ONL in the region likely contribute to this result. Parikh et al. [30] reported that age was related to the loss of neurons or glial cells in the inner retina, which may be responsible for the SD-OCT–examination outcome in this area.

Several studies have shown that assessment of GCL thickness could be a surrogate method to evaluate glaucomatous damage [31]. In this study, the observed age-related variation in GCL thickness in this ophthalmologically healthy cohort is a reminder that GCL thinning requires more accurate quantification before widespread adoption as a surrogate for glaucoma assessment.

The thickness of RPE in the pericentral and peripheral regions was significantly increased with aging. Many pathological changes led to the thickening of the RPE, which included the density of residual bodies and accumulation of lipofuscin, accumulation of basal deposits on or within the Bruch’s membrane, formation of drusen, and thickening of the Bruch’s membrane [32]. The age-related variation of RPE in the macular region requires future investigation.

This study has some limitations. The small sample size might have introduced some bias. Our data are limited to Chinese ethnicity and need to be tested in other ethnic groups in the future.

## Conclusions

Using SD-OCT, we assessed age-related thinning of ONL, RPE, and choroidal layers accompanied with thickened RNFL, INL, and OPL of the fovea in an ophthalmologically healthy Chinese cohort. The variations of individual layers in the fovea may be related to age-independent RT. It is speculated that the age-related reductions of RT in the pericentral and peripheral rings were associated with age-related thinning of GCL, IPL and ONL in these regions. Regular monitoring of the macular architecture using SD-OCT in ophthalmologically healthy people, especially among the aged population, should be considered in future evaluations.

## Appendix

**Table 6:** Mean Thickness of Retina and eight individual retinal layers in nine Macular Sectors

Retinal Layer	fovea	Pericentral Superior	Pericentral Nasal	Pericentral Inferior	Pericentral Temporal	Peripheral Superior	Peripheral Nasal	Peripheral Inferior	Peripheral Temporal
RT	257.04±18.85	339.37±17.56	339.58±17.22	335.10±16.97	324.92±15.82	300.39±15.80	317.43±21.96	287.16±15.33	283.77±15.41
RNFL	10.78±2.24	23.44±3.68	20.11±2.32	24.50±3.10	16.82±1.33	37.52±5.14	46.12±6.85	38.93±5.70	19.21±8.37
GCL	12.31±3.20	52.03±5.76	50.12 ±6.04	51.33±5.33	46.66±5.51	36.58±3.88	40.48±4.26	34.03±3.69	36.92±4.74
IPL	18.28±2.85	41.18±3.82	42.34±3.84	40.90±3.67	41.01±3.64	29.67±2.96	31.20±3.19	27.81±3.06	32.61±3.00
INL	15.27±4.63	39.75±4.47	38.48±4.09	40.46±4.23	36.48±3.84	32.33±3.19	35.07±3.03	32.09±3.34	33.90±2.63
OPL	23.48±6.08	32.86±8.44	31.87±8.27	36.17±9.90	31.14±6.02	26.15±2.91	28.20±3.47	27.02±3.12	26.37±2.58
ONL	88.44±11.40	67.48±12.72	72.52±11.54	60.45±12.68	70.02±9.31	58.15±7.73	55.87±7.57	49.58±7.16	55.80±6.33
PRL+RPE	90.19±4.40	82.74±3.15	83.94±3.27	81.32±3.26	82.60±3.12	79.80±3.22	79.84±2.69	77.81±2.84	79.84±2.69
RPE	17.5±2.20	16.16±1.89	16.30±1.99	15.36±1.76	15.10±1.60	14.29±1.37	14.18±1.47	13.70±1.76	13.50±1.38

Values are mean ± SD (μm). RT, retinal thickness; RNFL, retinal nerve fiber layer; GCL, ganglion cell layer; IPL, inner plexiform layer; INL, inner nuclear layer; OPL, outer plexiform layer; ONL, outer nuclear layer; PRL, photoreceptor layer; RPE, retinal pigment epithelium.

## Abbreviations

AMD: Age-related macular degeneration
BCVA: Best corrected visual acuity
BM: Bruch’s membrane
CSCR: Central serous chorioretinopathy
EDI: Enhanced-depth imaging
ELM: External limiting membrane
ETDRS: Early Treatment Diabetic Retinopathy Study
GCL: Ganglion cell layer
ILM: internal limiting membrane
INL: Inner nuclear layer
IOP: Intraocular pressure
IPL: Inner plexiform layer
IRT: Inner retinal thickness
OCT: Optical coherence tomography
ONL: Outer nuclear layer
OPL: Outer plexiform layer
PRL: Photoreceptor layer
RNFL: Retinal nerve fiber layer
RPE: Retinal pigment epithelium
RT: Retinal thickness
SD-OCT: Spectral-domain optical coherence tomography
SFCT: Subfoveal choroidal thickness

## References

[1] Swanson EA, Izatt JA, Hee MR, Huang D, Lin CP, Schuman JS, Puliafito CA, Fujimoto JG (1993). In vivo retinal imaging by optical coherence tomography. Opt Lett.

[2] Wang J, Gao X, Huang W, Wang W, Chen S, Du S, Li X, Zhang X (2015). Swept-source optical coherence tomography imaging of macular retinal and choroidal structures in healthy eyes. BMC Ophthalmol.

[3] Grover S, Murthy RK, Brar VS, Chalam KV (2010). Comparison of retinal thickness in normal eyes using stratus and Spectralis optical coherence tomography. Invest Ophthalmol Vis Sci.

[4] Hirasawa H, Tomidokoro A, Araie M, Konno S, Saito H, Iwase A, Shirakashi M, Abe H, Ohkubo S, Sugiyama K (2010). Peripapillary retinal nerve fiber layer thickness determined by spectral-domain optical coherence tomography in ophthalmologically normal eyes. Arch Ophthalmol.

[5] Huang W, Wang W, Zhou M, Chen S, Gao X, Fan Q, Ding X, Zhang X (2013). Peripapillary choroidal thickness in healthy Chinese subjects. BMC Ophthalmol.

[6] Spaide RF, Koizumi H, Pozzoni MC (2008). Enhanced depth imaging spectral-domain optical coherence tomography. Am J Ophthalmol.

[7] Lang A, Carass A, Hauser M, Sotirchos ES, Calabresi PA, Ying HS, Prince JL (2013). Retinal layer segmentation of macular OCT images using boundary classification. Biomed Opt Express.

[8] Matsumoto H, Sato T, Kishi S (2009). Outer nuclear layer thickness at the fovea determines visual outcomes in resolved central serous chorioretinopathy. Am J Ophthalmol.

[9] Sun JK, Lin MM, Lammer J, Prager S, Sarangi R, Silva PS, Aiello LP (2014). Disorganization of the retinal inner layers as a predictor of visual acuity in eyes with center-involved diabetic macular edema. JAMA Ophthalmol.

[10] Chung YR, Kim JW, Choi SY, Park SW, Kim JH, Lee K. Subfoveal Choroidal Thickness And Vascular Diameter In Active And Resolved Central Serous Chorioretinopathy. Retina. 2018;38:102–107. 10.1097/IAE.0000000000001502 28106708

[11] Zucchiatti I, Parodi MB, Pierro L, Cicinelli MV, Gagliardi M, Castellino N, Bandello F. Macular ganglion cell complex and retinal nerve Fiber layer comparison in different stages of age-related macular degeneration. Am J Ophthalmol. 2015;160:602–607 e601. 10.1016/j.ajo.2015.05.030 26052088

[12] Chirco KR, Sohn EH, Stone EM, Tucker BA, Mullins RF (2017). Structural and molecular changes in the aging choroid: implications for age-related macular degeneration. Eye (Lond).

[13] Garcia-Martin E, Polo V, Larrosa JM, Marques ML, Herrero R, Martin J, Ara JR, Fernandez J, Pablo LE (2014). Retinal layer segmentation in patients with multiple sclerosis using spectral domain optical coherence tomography. Ophthalmology.

[14] Sari ES, Koc R, Yazici A, Sahin G, Ermis SS. Ganglion cell-inner plexiform layer thickness in patients with Parkinson disease and association with disease severity and duration. J Neuroophthalmol. 2015;35:117–21. 10.1097/WNO.0000000000000203 25485861

[15] Liu D, Zhang L, Li Z, Zhang X, Wu Y, Yang H, Min B, Zhang X, Ma D, Lu Y (2015). Thinner changes of the retinal nerve fiber layer in patients with mild cognitive impairment and Alzheimer’s disease. BMC Neurol.

[16] Peng PH, Lin HS, Lin S (2009). Nerve fibre layer thinning in patients with preclinical retinopathy. Can J Ophthalmol.

[17] Song WK, Lee SC, Lee ES, Kim CY, Kim SS (2010). Macular thickness variations with sex, age, and axial length in healthy subjects: a spectral domain-optical coherence tomography study. Invest Ophthalmol Vis Sci.

[18] Ooto S, Hangai M, Yoshimura N (2015). Effects of sex and age on the normal retinal and choroidal structures on optical coherence tomography. Curr Eye Res.

[19] Eriksson U, Alm A (2009). Macular thickness decreases with age in normal eyes: a study on the macular thickness map protocol in the stratus OCT. Br J Ophthalmol.

[20] Alasil T, Wang K, Keane PA, Lee H, Baniasadi N, de Boer JF, Chen TC (2013). Analysis of normal retinal nerve fiber layer thickness by age, sex, and race using spectral domain optical coherence tomography. J Glaucoma.

[21] Ikuno Y, Kawaguchi K, Nouchi T, Yasuno Y (2010). Choroidal thickness in healthy Japanese subjects. Invest Ophthalmol Vis Sci.

[22] Lim MC, Hoh ST, Foster PJ, Lim TH, Chew SJ, Seah SK, Aung T (2005). Use of optical coherence tomography to assess variations in macular retinal thickness in myopia. Invest Ophthalmol Vis Sci.

[23] Demirkaya N, van Dijk HW, van Schuppen SM, Abramoff MD, Garvin MK, Sonka M, Schlingemann RO, Verbraak FD (2013). Effect of age on individual retinal layer thickness in normal eyes as measured with spectral-domain optical coherence tomography. Invest Ophthalmol Vis Sci.

[24] Grover S, Murthy RK, Brar VS, Chalam KV (2009). Normative data for macular thickness by high-definition spectral-domain optical coherence tomography (spectralis). Am J Ophthalmol.

[25] Huang D, Swanson EA, Lin CP, Schuman JS, Stinson WG, Chang W, Hee MR, Flotte T, Gregory K, Puliafito CA (1991). Optical coherence tomography. Science.

[26] Rogala J, Zangerl B, Assaad N, Fletcher EL, Kalloniatis M, Nivison-Smith L (2015). In vivo quantification of retinal changes associated with drusen in age-related macular degeneration. Invest Ophthalmol Vis Sci.

[27] La Torre G, Pacella E, Saulle R, Giraldi G, Pacella F, Lenzi T, Mastrangelo O, Mirra F, Aloe G, Turchetti P (2013). The synergistic effect of exposure to alcohol, tobacco smoke and other risk factors for age-related macular degeneration. Eur J Epidemiol.

[28] Ooto S, Hangai M, Tomidokoro A, Saito H, Araie M, Otani T, Kishi S, Matsushita K, Maeda N, Shirakashi M (2011). Effects of age, sex, and axial length on the three-dimensional profile of normal macular layer structures. Invest Ophthalmol Vis Sci.

[29] Sung KR, Wollstein G, Bilonick RA, Townsend KA, Ishikawa H, Kagemann L, Noecker RJ, Fujimoto JG, Schuman JS (2009). Effects of age on optical coherence tomography measurements of healthy retinal nerve fiber layer, macula, and optic nerve head. Ophthalmology.

[30] Parikh RS, Parikh SR, Sekhar GC, Prabakaran S, Babu JG, Thomas R (2007). Normal age-related decay of retinal nerve fiber layer thickness. Ophthalmology.

[31] Distante P, Lombardo S, Verticchio Vercellin AC, Raimondi M, Rolando M, Tinelli C, Milano G (2015). Structure/function relationship and retinal ganglion cells counts to discriminate glaucomatous damages. BMC Ophthalmol.

[32] Bonilha VL (2008). Age and disease-related structural changes in the retinal pigment epithelium. Clin Ophthalmol.

